# Taxonomic revision of the Malagasy *Aphaenogaster swammerdami* group (Hymenoptera: Formicidae)

**DOI:** 10.7717/peerj.10900

**Published:** 2021-03-02

**Authors:** Sandor Csősz, Ana C. Loss, Brian L. Fisher

**Affiliations:** 1 Evolutionary Ecology Research Group, Institute of Ecology and Botany, Centre for Ecological Research, Vácrátót, Hungary; 2 National Institute of the Atlantic Forest, Santa Teresa, Brazil; 3 Entomology, California Academy of Sciences, San Francisco, CA, USA

**Keywords:** Biodiversity, Entomology, Taxonomy, Morphometry, Biogeography, New species, Phylogeny

## Abstract

**Background:**

Madagascar is famous for its extremely rich biodiversity; the island harbors predominantly endemic and threatened communities meriting special attention from biodiversity scientists. Continuing ongoing efforts to inventory the Malagasy ant fauna, we revise the species currently placed in the myrmicine genus *Aphaenogaster* Mayr. One species described from Madagascar, *Aphaenogaster friederichsi* Forel, is synonymized with the Palearctic *A. subterranea* Latreille **syn. nov.** This species is considered neither native to Madagascar nor established in the region. This revision focuses on the balance of species in the *A. swammerdami* group which are all endemic to Madagascar.

**Methods:**

The diversity of the Malagasy *Aphaenogaster* fauna was assessed via application of multiple lines of evidence involving quantitative morphometric, qualitative morphological, and DNA sequence data. (1) Morphometric investigation was based on hypothesis-free *Nest Centroid clustering* (NC-clustering) combined with *PArtitioning based on Recursive Thresholding* (PART) to estimate the number of morphological clusters and determine the most probable boundaries between them. This protocol provides a repeatable and testable approach to find patterns in continuous morphometric data. Species boundaries and the reliability of morphological clusters recognized by these exploratory analyses were tested via confirmatory Linear Discriminant Analysis (LDA). (2) Qualitative, external morphological characteristics (e.g., shape, coloration patterns, setae number) were subjectively evaluated in order to create a priori grouping hypotheses, and confirm and improve species delimitation. (3) Species delimitation analyses based on mitochondrial DNA sequences from cytochrome oxidase I (COI) gene fragments were carried out to test the putative species previously delimited by morphological and morphometric analyses.

**Results:**

Five species can be inferred based on the integrated evaluation of multiple lines of evidence; of these, three are new to science: *Aphaenogaster bressleri* **sp. n**., *A. gonacantha* (Emery, 1899), *A. makay* **sp. n.**, *A. sahafina* **sp. n.**, and *A. swammerdami* Forel, 1886. In addition, three new synonymies were found for *A. swammerdami* Forel, 1886 (*A. swammerdami clara* Santschi, 1915 **syn. n.**, *A. swammerdami curta* Forel, 1891 **syn. n.** and *A. swammerdami spinipes* Santschi, 1911 **syn. n.**). Descriptions and redefinitions for each taxon and an identification key for their worker castes using qualitative traits and morphometric data are given. Geographic maps depicting species distributions and biological information regarding nesting habits for the species are also provided.

## Introduction

The Malagasy zoogeographical region is known to harbor extremely diverse and unique biota, and has recently been the focus of extensive biodiversity and systematic research (Fisher, 2009; Hita Garcia & Fisher, 2014; Csősz & Fisher, 2015). Beyond understanding the history of the region and the evolution of the fauna and ecological processes, accurate information on the Malagasy fauna is indispensable for creating plans to halt extinction in this highly vulnerable region. These efforts to explore Malagasy biodiversity has considerably increased our knowledge of the island’s myrmecofauna. The latest findings report extremely high species diversity in many genera of the region. The goal of the current paper is to contribute to this endeavor and prepare a species-level taxonomic revision of the myrmicine genus *Aphaenogaster* Mayr on Madagascar.

The native Malagasy *Aphaenogaster* species were either first described or combined in the genus or subgenus *Deromyrma* Forel. The type species of *Deromyrma*, *A. swammerdami* Forel, 1886, has been included in a number of molecular phylogenetic studies of ants. These studies have consistently shown that *Aphaenogaster* is not monophyletic and that *swammerdami* is of a different lineage than *Aphaenogaster* sensu stricta (Brady et al., 2006; Moreau, 2006; Moreau & Bell, 2013; Ward et al., 2015; Demarco & Cognato, 2015; DeMarco & Cognato, 2016).

Here we refer to the native species on Madagascar as members of the *A. swammerdami* group even though evidence indicates that they represent a different lineage from *Aphaenogaster* sensu stricta. Though placing the native species in a separate genus would more accurately reflect the different evolutionary history of the Malagasy species, we refrain from classifying them in the first available genus group name *Deromyrma* at this time. Though all members of Malagasy *swammerdami* group would belong to this clade, it is not clear which members from outside the Malagasy region would also belong. To address this point, future morphological and molecular studies would need to include species from Central America, Australasia, Indomalaya, and the southern Palearctic regions.

On Madagascar, the *swammerdami-*group species are abundant and dominant in many habitats. The genus includes some of the largest ant species in the region, which typically nest in soil or hollow wood cavities in lower vegetation. Foragers can also be found alone on the ground, in leaf litter, or in debris. The most striking morphological feature of this group is the presence of a cephalic constriction that forms a conspicuous “neck” on the posterior portion of the head capsule. The neck is present in workers, queens and males. Propodeal spines may be short to moderately long, but are always present, while legs and antennae, including scapes, are also elongated.

*Aphaenogastaer swammerdami* is one of the few ant species in Madagascar that has been the target of ecological studies, in particular at Kirindy Forest (CNFEREF), a dry deciduous forest in central western Madagascar. Studies have shown that colony density and size differ depending on level of disturbance and logging (Tornyie & Borketey-La, 2013; Dittmann, Dammhahn & Kappeler, 2014). Böhning-Gaese, Burkhardt & Schmid (1996), Böhning-Gaese, Gaese & Rabemanantsoa (1999) and Voigt et al. (2002) suggest that this species plays an important role in the secondary dispersal of seeds of the Malagasy tree *Commiphora guillaumini* and seeds in general.

We assess the diversity of the Malagasy *Aphaenogaster* via an integrated taxonomic protocol incorporating morphometrics, morphology, and DNA sequence data. Quantitative analyses of continuous morphometric traits were performed via NC-PART clustering described by Csősz & Fisher (2016a, 2016b). This method incorporates two multivariate approaches: (1) NC-clustering (Seifert, Ritz & Csősz, 2014) designed to find structure in large sets of continuous morphometric data and (2) partitioning algorithms known as “part” (Nilsen et al., 2013) that assign cases into clusters and determine the ideal number of clusters via gap statistic algorithms (Tibshirani, Walther & Hastie, 2001). The clusters found via this protocol are considered species unless this hypothesis conflicts with other biological information. Species boundaries and the reliability of morphological clusters recognized by these exploratory analyses were tested via confirmatory Linear Discriminant Analysis (LDA), cross validation (LOOCV), subjective evaluation of qualitative morphological characteristics, and DNA-based species delimitation analysis.

We used the stopping rule (Schlick-Steiner et al., 2010)—where species are described if morphometric, subjectively evaluated external morphological data, and molecular approaches agree in classification—for all save one species. For *A. makay*, besides the clear molecular and qualitative morphological separation, the few available samples were below the threshold level to apply the quantitative, morphometry based cluster delimitation protocol (PART). The description of *A. makay* was supported by agreement in qualitative morphological characteristics and molecular data.

According to the combined application of the analyses, a total of five species are inferred. Three of these (*Aphaenogaster bressleri*, *A. makay*, *and A. sahafina*) are described as new species in addition to two previously described species, *A. gonacantha* (Emery, 1899), and *A. swammerdami* Forel, 1886. Three subspecies are also synonymized with *A. swammerdami* Forel, 1886: *A. swammerdami clara* Santschi, 1915, *A. swammerdami curta* Forel, 1891 and *A. swammerdami spinipes* Santschi, 1911.

## Materials and Methods

In the present study, 16 continuous morphometric traits were recorded in 176 worker individuals from 96 collecting events in Madagascar. Specimens evaluated for this study were from the following institutions: CASC (California Academy of Sciences, San Francisco, CA, USA), MHNG (Muséum d’Histoire Naturelle, Geneva, Switzerland), MSNG (Museo Civico di Storia Naturale “Giacomo Doria”, Genova, Italy), NHMB (Naturhistorisches Museum, Basel, Switzerland), PSWC (Phil S. Ward’s collection, University of California Davis, Davis, CA, USA). Type material and samples that were morphometrically investigated are presented in the “Type material investigated” and “Material examined” sections in the following format: collection code (in bold), unique identifying CASENT code, verbatim locality, longitude, latitude, elevation in meters, collector, date in MM.DD.YYYY (number of individuals measured, abbreviation of depository). Additional information on habitat and microhabitat is discussed in the Distribution and Biology sections for each species. Raw data, including indices and classification results for material morphometrically examined in this work, is given in Table S1.

All specimen information and relevant images used in this study are available online on AntWeb (antweb.org). The images were taken by staff at the California Academy of Sciences and the person taking the images is provided in the figure caption. The images are copyrighted by the California Academy of Sciences and licensed under a Creative Commons Attribution 4.0 International (CC BY 4.0).

Digital color montage images were created using a JVC KY-F75 digital camera and Syncroscopy Auto-Montage software (version 5.0), or a Leica DFC 425 camera in combination with the Leica Application Suite software (version 3.8). Distribution maps were generated in R (R Development Core Team, 2015) via the “phylo.to.map” function using package phytools (Revell, 2012).

### Morphometric character recording

Measurements were taken with a Leica M165C stereomicroscope equipped with an ocular micrometer at a magnification of ×25 to ×100. Body size dimensions are expressed in µm. Due to rarity or lack of queen and male specimens, the present revision is based only on the worker caste. All measurements were made by SC. Abbreviations of morphometric traits, explanations for measured characters, and the magnification applied for each certain trait, are given in Table 1. The complex morphometry-based statistical framework, including hypothesis formation and testing, follows the protocol published in detail in Csősz & Fisher (2016a, 2016b).

**Table 1:** Abbreviation (Abbr.) of morphometric characters, definition of measurements, and magnification (Mag.) used to measure each certain trait.

Abbr.	Description of the trait	Mag.
CL	Maximum median length of head capsule. The head must be carefully tilted so the maximum length is positioned in the measuring plane	×50
CWb	Maximum width of head capsule in full-face view	×50
EL	Eye length. Maximum diameter of the compound eye	×100
FL	Maximum width of frontal lobes	×100
FRS	Frontal carina distance. Distance of the frontal carinae immediately caudal of the posterior intersection points between the frontal carinae and the torular lamellae	×100
ML	Diagonal length of the alitrunk in profile. Measured in lateral view from the anteriormost point of anterior pronotal slope to the caudalmost point of the lateral metapleural lobe	×25
MW	Maximum width of pronotum	×100
NOH	Petiole node height; in a right angle from a reference line beginning at the petiolar spiracle and ending at the most caudo-dorsal point of the petiole	×100
PEL	Petiole length; horizontal distance between the petiolar spiracle and the most caudo-dorsal point of the petiole	×100
PEW	Petiole width. The maximum width of petiole in dorsal view	×100
PoOC	Postocular distance. Use a cross-scaled ocular micrometer and adjust the head to the measuring position of CL. Caudal measuring point: median occipital margin; frontal measuring point: reference line between the caudalmost border of the two compound eyes	×50
PPH	Postpetiole height. Maximum height of the postpetiole in lateral view. Measured perpendicularly to a line defined by the linear section of the segment border between dorsal and ventral petiolar sclerite	×100
PPW	Postpetiole width. Maximum width of postpetiole in dorsal view	×100
SL	Scape length. The maximum straight-line scape length excluding the articular condyle	×25
SPST	Propodeal spine length. Distance between the center of propodeal stigma and spine tip	×100
SPTI	Propodeal spine apical distance. The distance between spine tips in dorsal view; if spine tips are rounded or truncated, the centers of spine tips are taken as reference points	×100

### Qualitative morphology-based species delimitation

This workflow involves external morphological characteristics regularly considered in conventional verbatim species descriptions. Shapes, color patterns, setae number, and sculpture characteristics were subjectively evaluated during this work phase. A Leica LED3000 SLI LED Spotlight Illuminator with gooseneck cold-light source equipped with two flexible, focally mounted light-cables were used to characterize color patterns.

### DNA based-species delimitation analysis

We analyzed 658 base pairs (bp) of the mitochondrial cytochrome oxidase I (COI) gene from 89 Malagasy *Aphaenogaster* specimens (Appendix 1). DNA was extracted using specimen legs, allowing preservation of vouchers. DNA extraction and COI sequencing were performed at the University of Guelph (Ontario, Canada), following the protocol described in Fisher & Smith (2008). All sequences are available at GenBank and BOLD (Table S2). We also included 2 sequences from GenBank as outgroup, *Cephalotes texanus* (KC335803.1) and *Tapinoma magnum* (KY426518.1). Sequences were aligned using Geneious 11.1.5 (Biomatters Ltd., Auckland, New Zealand; Masters, Fan & Ross, 2011). Final alignment is available as Supplemental File S3. To exclude redundancies in the matrix, we removed duplicated haplotypes using the Alter (Glez-Peña et al., 2010) online platform (http://sing.ei.uvigo.es/ALTER/). The final simplified data matrix had 74 sequences consisting of 72 unique *Aphaenogaster* haplotypes and 2 outgroups.

PartitionFinder2 (Lanfear et al., 2017) was used to establish the best nucleotide substitution model and partition scheme for the data under the corrected Akaike Information Criteria (AICc). Models that estimated a proportion of invariant sites (“+I” parameter) were not considered to avoid overparameterization when selecting the gamma shape parameter (“+G”) (Mayrose, Friedman & Pupko, 2005; Stamatakis, 2006). The best fit model selected for each codon position (1 + 2 + 3) was GTR+G. Bayesian phylogenetics were estimated in BEAST v2.5.1 (Bouckaert et al., 2014) using a strict molecular clock, empirical frequency base, nucleotide substitution model, and codon partition established by PartionFinder2. Analyses ran for 10^9^ generations, with one tree sampled every 10^5^ generation, resulting in 10^4^ trees. MCMC chain stationarity and likelihood ESS values greater than 200 were verified in Tracer 1.7.1 (Rambaut et al., 2018). Consensus tree was computed in TreeAnotator 2.5.1 (BEAST package) after discarding the first 10% of trees as burn-in.

### Species delimitation

Bayesian consensus tree was used as input to test the putative species designated a priori based on morphological and morphometric data. Analyses were conducted with the Species Delimitation Plugin (SDP; Masters, Fan & Ross, 2011), implemented in Geneious, using the following metrics: (i) monophyly; (ii) average intraspecific uncorrected pairwise distance (Intra Dist); (iii) average uncorrected pairwise distance between a putative species and its sister species (Inter Dist); (iv) PID Liberal (Ross, Murugan & Li, 2008); and (v) Rosenberg’s P_AB_ statistics (Rosenberg, 2007).

PID Liberal measures the probability of a correct identification of an unknown specimen as a member of the putative species or its sister species, while Rosenberg’s P_AB_ is the probability of reciprocal monophyly by random chance. Thus, we expect valid species to be monophyletic, with overall intraspecific distance smaller than interspecific, high PID Liberal values, and small values of Rosenberg’s P_AB_.

### Field study permissions

The following information was supplied relating to field study approvals (i.e., approving body and any reference numbers): Ant samples used in this study comply with the regulations for export and exchange of research samples outlined in the Convention of Biology Diversity and the Convention on International Trade in Endangered Species of Wild Fauna and Flora. For field work conducted in Madagascar, permits to research, collect, and export ants were obtained from the Ministry of Environment and Forest as part of an ongoing collaboration between the California Academy of Sciences and the Ministry of Environment and Forest, Madagascar National Parks, and Parc Botanique et Zoologique de Tsimbazaza. Authorization for export was provided by the Director of Natural Resources.

Approval Numbers:

No. 0142N/EA03/MG02,

No. 340N-EV10/MG04,

No. 69 du 07/04/06,

No. 065N-EA05/MG11,

No. 047N-EA05/MG11,

No. 083N-A03/MG05,

No. 206 MINENVEF/SG/DGEF/DPB/SCBLF,

No. 0324N/EA12/MG03,

No. 100 l/fEF/SG/DGEF/DADF/SCBF,

No. 0379N/EA11/MG02,

No. 200N/EA05/MG02.

### New Zoological Taxonomic Names

The electronic version of this article in Portable Document Format (PDF) will represent a published work according to the International Commission on Zoological Nomenclature (ICZN), and hence the new names contained in the electronic version are effectively published under that Code from the electronic edition alone. This published work and the nomenclatural acts it contains have been registered in ZooBank, the online registration system for the ICZN. The ZooBank LSIDs (Life Science Identifiers) can be resolved and the associated information viewed through any standard web browser by appending the LSID to the prefix http://zoobank.org/. The LSID for this publication is: urn:lsid:zoobank.org:pub:A66CA7F2-7334-40CA-A368-E522BA54ACBF. The online version of this work is archived and available from the following digital repositories: PeerJ, PubMed Central and CLOCKSS.

## Results and Discussion

**Figure 1:**
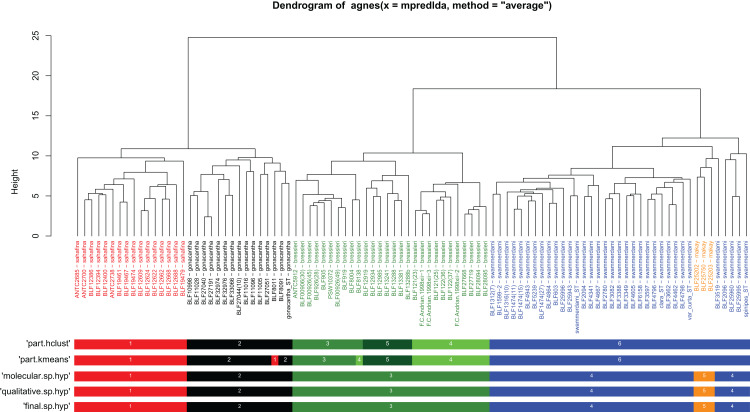
Quantitative morphometry-based dendrogram solution for Malagasy *Aphaenogaster* species. Specimen labels in the dendrogram follows this format: final species hypothesis followed by collection code number separated by a hyphen. The grouping labels (color bars) represent results of four methods: partitioning resulted by method PART using two cluster methods ‘hclust’ and “kmeans”, molecular hypothesis based on CO1, and the qualitative morphology-based hypothesis. Final species hypothesis bar shows classification of samples after confirmation by cross-validated LDA. Different colors distinguish species. *Aphaenogaster bressleri* **sp. n.**: green, *A. gonacantha* (Emery, 1899): black, *A. sahafina* **sp. n.**: red, *A. swammerdami* Forel, 1886: blue, *A. makay* **sp. n.**: orange.

Both clustering methods “hclust” and “kmeans” using function “part” based on NC-clustering returned six clusters (Fig. 1). Five clusters were confirmed via subjective evaluation of qualitative traits and mtDNA; three clusters recognized by NC-PART clustering (*A. bressleri*, shown by green bars in Fig. 1) were not convincingly supported via Bayesian phylogeny using mtDNA data, hence these three clusters were lumped and described as a single species, *A. bressleri*. Another cluster of specimens (marked by orange bars in Fig. 1) that was not recognized via NC-PART clustering was outlined via descriptive traits and mtDNA. This cluster of specimens possesses a unique combination of traits that would qualify for a species description, but the small number of samples (a total of 3 workers were available) were below the minimum cluster size threshold (minsize = 5) set for the clustering methods. Hence this cluster—though their position is somewhat separated from the bulk of the *swammerdami* cluster—remained unrecognized by the gap statistics. We added this species, *A. makay*, to our morphological species hypothesis as a recognized entity.

**Figure 2:**
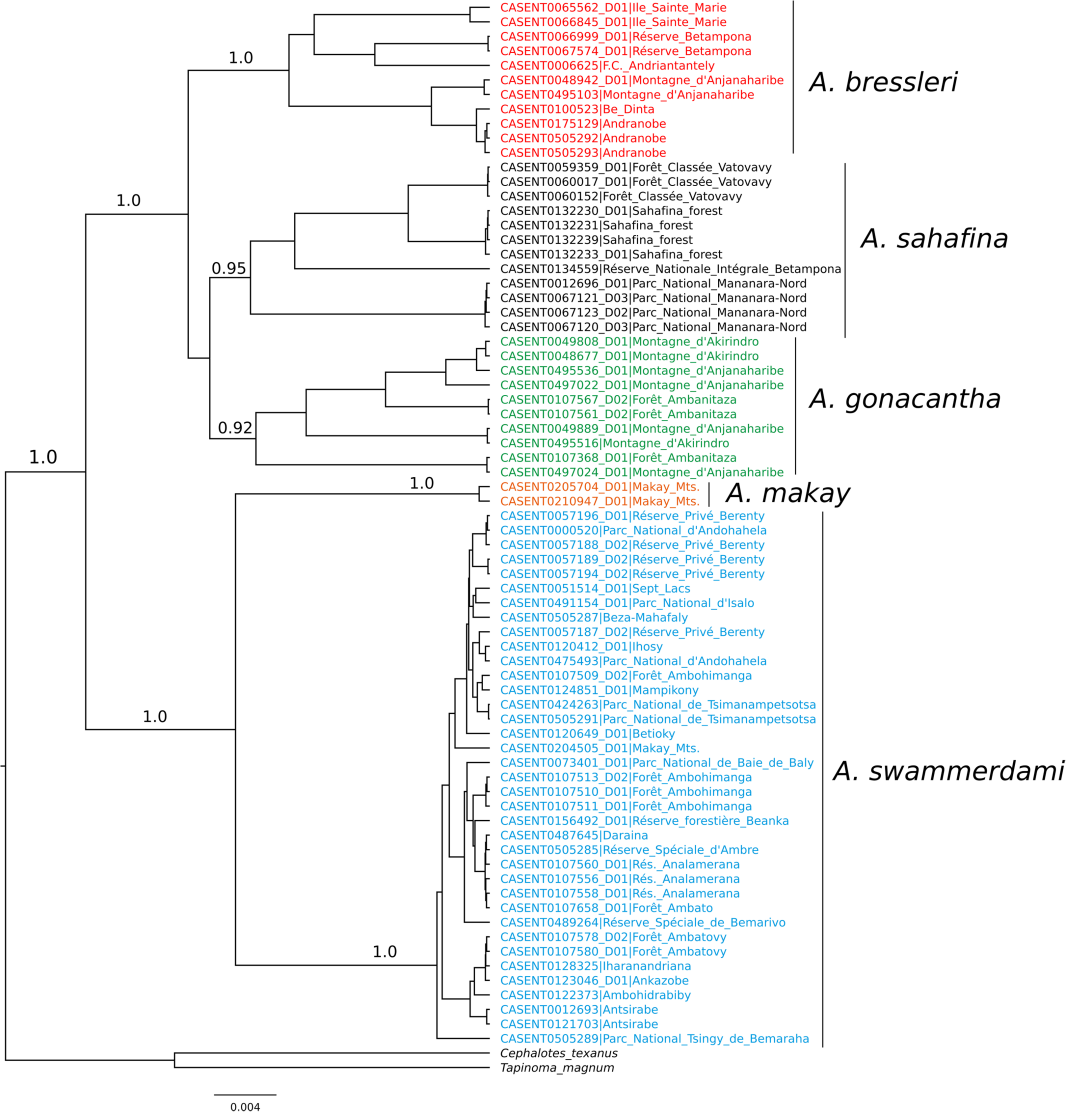
Bayesian phylogeny of *Aphaenogaster* COI sequences. Values associated with nodes correspond to Bayesian posterior probability.

When the five-species hypothesis was tested by LDA and by LOOCV, the overall classification success was 100%. All putative species with COI sequences available also showed satisfactory results for all DNA species delimitation criteria used (Fig. 2; Table 2). All clusters proved to be monophyletic, with intraspecific distance varying from 0.1% (*A. makay*) to 2.0% (*A. gonacantha* and *A. sahafina*) falling outside the range of interspecific divergence (from 3.6% to 3.9%), average PID Liberal values ranging from 0.91 (*A. gonacantha*) to 0.98 (*A. swammerdami*) and Rosenberg’s P_AB_ values lower than 0.0001.

**Table 2:** Results from species delimitation plugin analyses for *Aphaenogaster* putative species defined by morphological and morphometric analyses.

Species	Monophyletic	Intra Dist (%)	Inter Dist Closest (%)	PID Liberal (95% CI)	Rosenberg’s P_AB_
*A. sahafina*	yes	2.0	3.6	0.94 [0.90–0.98]	>0.0001
*A. gonacantha*	yes	2.0	3.6	0.91 [0.85–0.96]	>0.0001
*A. bressleri*	yes	1.9	3.9	0.92 [0.87–0.97]	>0.0001
*A. swammerdami*	yes	0.5	3.3	0.98 [0.95–1.0]	>0.0001
*A. makay*	yes	0.1	3.3	0.96 [0.81–1.0]	>0.0001

**Note:**

Average intraspecific pairwise distance (Intra Dist); average interspecific pairwise (Inter Dist); mean PID Liberal probability and its 95% confidence interval (95% CI); and Rosenberg’s PAB statistics. Refer to text for explanation of metrics.

We found complete agreement between monophyly of clusters in the molecular phylogeny and the quantitative morphology-based cluster delimitation protocols. Taking multiple lines of evidence into account, we describe the five clusters as five species: *Aphaenogaster bressleri*, *A. gonacantha* (Emery, 1899), *A. makay*, *A. sahafina*, and *A*. *swammerdami* Forel, 1886. These species also differ in qualitative diagnostic features and body ratios (for body ratios, see Table 3).

**Table 3:** Mean of morphometric ratios calculated for each species based on individuals.

	*Bressleri*	*Gonacantha*	*Makay*	*Sahafina*	*Swammerdami*
	(*n* = 53)	(*n* = 27)	(*n* = 3)	(*n* = 32)	(*n* = 62)
CWb	1,292 ± 70	1,453 ± 73	1,134 ± 97	1,295 ± 89	1,374 ± 147
	[1,156–1,450]	[1,300–1,580]	[1,036–1,230]	[1,064–1,430]	[1,030–1,850]
CL/CWb	1.79 ± 0.04	1.75 ± 0.04	1.72 ± 0.03	1.76 ± 0.05	1.61 ± 0.07
	[1.69–1.90]	[1.63–1.80]	[1.68–1.74]	[1.65–1.88]	[1.44–1.78]
POOC/CWb	0.98 ± 0.03	0.97 ± 0.03	0.88 ± 0.02	0.99 ± 0.03	0.80 ± 0.04
	[0.93–1.06]	[0.87–1.02]	[0.87–0.90]	[0.92–1.05]	[0.70–0.87]
FRS/CWb	0.23 ± 0.01	0.24 ± 0.02	0.25 ± 0.01	0.24 ± 0.02	0.24 ± 0.01
	[0.21–0.26]	[0.21–0.28]	[0.24–0.25]	[0.21–0.28]	[0.21–0.28]
FL/CWb	0.32 ± 0.01	0.34 ± 0.01	0.33 ± 0.01	0.34 ± 0.01	0.33 ± 0.01
	[0.30–0.35]	[0.31–0.36]	[0.32–0.34]	[0.32–0.36]	[0.29–0.35]
SL/CWb	2.36 ± 0.06	2.25 ± 0.07	2.22 ± 0.07	2.30 ± 0.08	1.92 ± 0.12
	[2.25–2.59]	[2.10–2.38]	[2.15–2.27]	[2.17–2.45]	[1.62–2.16]
MW/CWb	0.77 ± 0.02	0.71 ± 0.01	0.78 ± 0.02	0.70 ± 0.02	0.74 ± 0.03
	[0.74–0.81]	[0.69–0.74]	[0.76–0.81]	[0.66–0.75]	[0.67–0.89]
SPTI/CWb	0.31 ± 0.06	0.45 ± 0.07	0.24 ± 0.01	0.40 ± 0.05	0.26 ± 0.03
	[0.17–0.43]	[0.30–0.57]	[0.24–0.25]	[0.32–0.58]	[0.16–0.35]
PEW/CWb	0.26 ± 0.01	0.26 ± 0.02	0.30 ± 0.01	0.25 ± 0.01	0.26 ± 0.01
	[0.22–0.28]	[0.24–0.28]	[0.29–0.30]	[0.21–0.27]	[0.23–0.30]
PPW/CWb	0.40 ± 0.02	0.39 ± 0.02	0.42 ± 0.05	0.38 ± 0.02	0.40 ± 0.02
	[0.38–0.45]	[0.35–0.42]	[0.37–0.46]	[0.34–0.41]	[0.35–0.46]
ML/CWb	2.44 ± 0.07	2.34 ± 0.05	2.37 ± 0.02	2.39 ± 0.07	2.24 ± 0.10
	[2.31–2.59]	[2.24–2.46]	[2.36–2.39]	[2.29–2.57]	[1.97–2.49]
SPST/CWb	0.45 ± 0.04	0.70 ± 0.04	0.22 ± 0.01	0.64 ± 0.04	0.33 ± 0.03
	[0.36–0.56]	[0.64–0.81]	[0.21–0.23]	[0.55–0.73]	[0.28–0.40]
NOL/CWb	0.50 ± 0.03	0.50 ± 0.02	0.54 ± 0.02	0.53 ± 0.02	0.47 ± 0.03
	[0.45–0.55]	[0.46–0.55]	[0.51–0.55]	[0.49–0.57]	[0.40–0.57]
NOH/CWb	0.22 ± 0.02	0.21 ± 0.01	0.28 ± 0.01	0.22 ± 0.02	0.25 ± 0.01
	[0.19–0.26]	[0.18–0.23]	[0.27–0.29]	[0.19–0.26]	[0.22–0.29]
PPH/CWb	0.39 ± 0.02	0.36 ± 0.02	0.44 ± 0.01	0.38 ± 0.02	0.40 ± 0.02
	[0.36–0.44]	[0.32–0.40]	[0.43–0.45]	[0.35–0.42]	[0.36–0.46]
EL/CWb	0.27 ± 0.02	0.25 ± 0.01	0.27 ± 0.01	0.25 ± 0.01	0.27 ± 0.01
	[0.25–0.30]	[0.27–0.29]	[0.24–0.26]	[0,26–0,28]	[0.23–0.28]

**Note:**

Morphometric traits are divided by cephalic size (CWb), ±SD are provided in the upper row, minimum and maximum values are given in parentheses in the lower row.

Classification of measured type specimens was set to wildcard in LDA; i.e., no grouping label was added for type specimens, as their classifications were predicted by the LDA that assigns posterior probabilities to each individual in the analysis reflecting the uncertainty of assessing an observation to a particular class. The geometric mean of posterior p values was calculated for syntype series. Type material (series of 2 workers) of *A. gonacantha* (Emery, 1899) was placed in a cluster named *A. gonacantha* (Emery, 1899), with posterior probability *p* = 0.988. The four other type materials, *A. swammerdami* Forel, 1886 (3 syntype workers), *A. swammerdami clara* Santschi, 1915 (single worker), *A. swammerdami curta* Forel, 1891 (2 syntype workers) and *A. swammerdami spinipes* Santschi, 1911 (single worker) were all placed in a morphological cluster named *A. swammerdami* Forel, 1886 with posterior probability *p* = 1.0, and we hereby propose the three subspecific names are junior synonyms of *A. swammerdami*.

**Figure 3:**
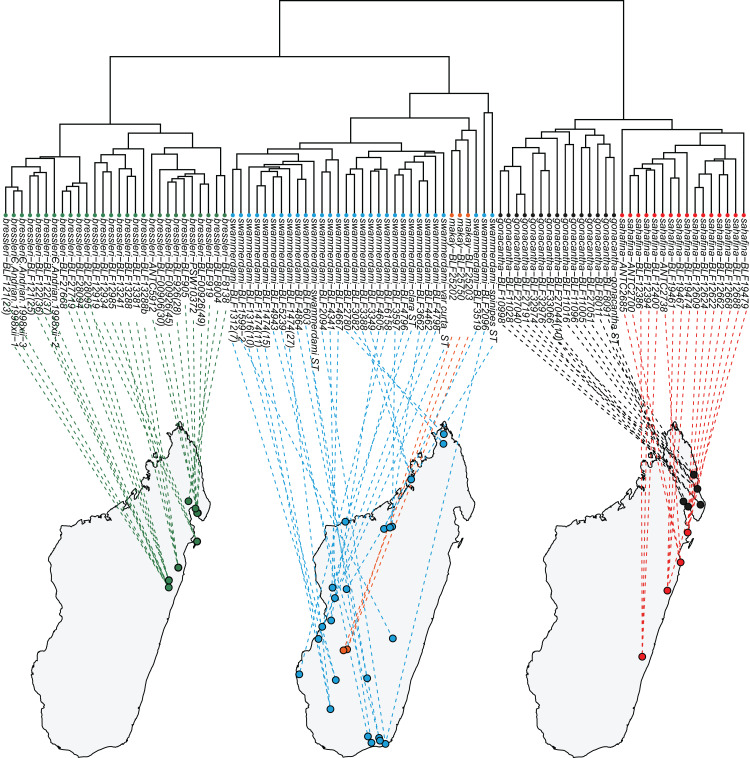
Dendrogram to geographic map. Dendrogram solution is linked to the map of Madagascar. Color codes for species are as follows: *Aphaenogaster bressleri* **sp. n.**: green, *A. gonacantha* (Emery, 1899): black, *A. sahafina* **sp. n.**: red, *A. swammerdami* Forel, 1886: blue, *A. makay* **sp. n.**: orange.

According to the material available, *A. bressleri*, *A. gonacantha* and *A. sahafina* are restricted to a north/south strip of the eastern humid forests of the island (Fig. 3). Only one species, *A. swammerdami*, can be considered widely distributed and extremely abundant in Madagascar, with a distribution stretching along the western coast and central part of the island but completely absent in eastern regions. One species, *A. makay*, is only known from humid forest in the Makay Massif. It was collected from bamboo forest on sandy soil at the headwaters of canyon streams at the base of cliffs.

### Malagasy *Aphaenogaster swammerdami*-group species

**Figure 4:**
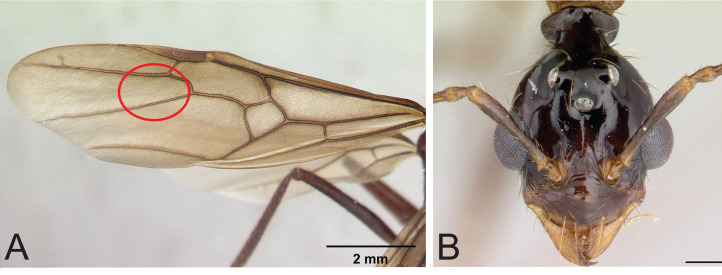
Identifying characters of *Aphaenogaster swammerdami* group. (A) Forewings lack 2rs-m: *Aphaenogaster swammerdami* queen CASENT0000523; (B) The neck and collar is present in *Aphaenogaster swammerdami* group males CASENT0000990. *A. swammerdami* male CASENT0000990. Image credit: California Academy of Sciences (https://www.antweb.org/specimenImages.do?code=CASENT0000523, https://www.antweb.org/specimenImages.do?code=CASENT0000990), CC-BY 4.0 International license.

Diagnosis within Madagascar. Among the Malagasy myrmicines, *Aphaenogaster swammerdami*-group species have 12-segmented antennae terminating in a weakly defined 4-segmented club or with apical 4 segments gradually increasing in size towards the apex. Queens and males are alate and characterized by the absence of the 2rs-m vein in the forewing of males and gynes (Fig. 4A). The posterior portion of the head capsule of workers, gynes, and males is always drawn out into a strongly constricted neck, behind which the head capsule flares out again to form a pronounced collar (Fig. 4B). The neck is less developed in some species but the collar is always present. Some Malagasy *Pheidole* species also have a neck and collar (e.g., *Pheidole grallatrix* Emery, 1899) but in these *Pheidole* the antenna terminates in a strongly defined three-segmented club while in the *swammerdami* group, the club is less apparent, four-segmented, and gradually increases in size towards the apex. The first gastral tergite (tergite of A4) broadly overlaps the sternite on the side of the gaster, and the sting is very reduced, never visible. The workers are large and elongate, with long, spindly legs. Workers and queens are dark red to black.

**Figure 5:**
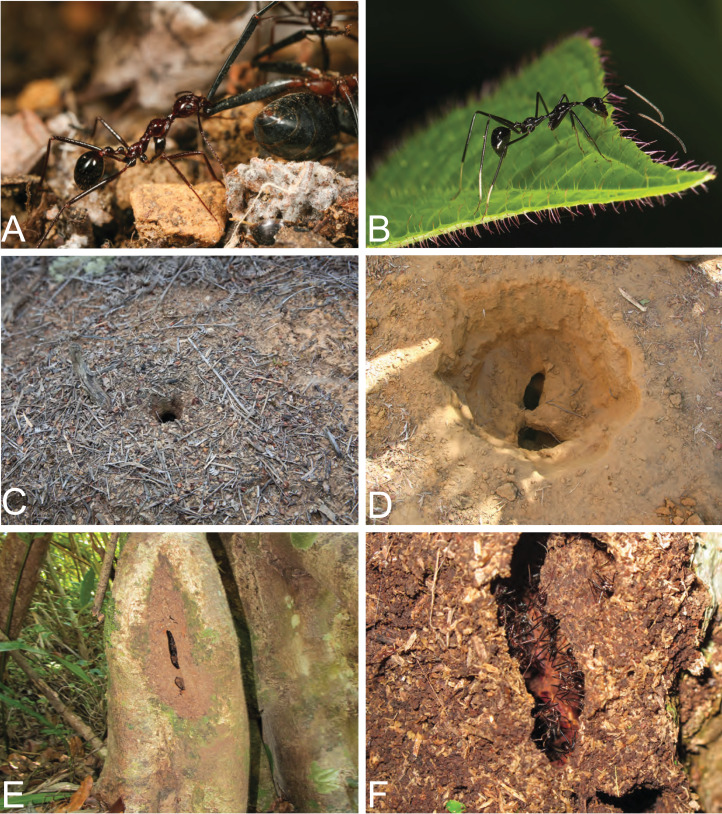
Life history traits of *Aphaenogaster*. (A) *A. swammerdami* dragging a dead *Camponotus* back to the nest in Kirinidy forest in Western Madagascar. (B) *Camponotus karaha*, one of the four *Camponotus* species that mimic *Aphaenogaster* in Madagascar. (C) *A. swarmmerami* ground nests are conspicuous. (D) The start of an excavation of an *A. swammerdami* nest in Kirindy Forest. (E) *A. gonocantha* nest in cavities closed with carton in Makirovana Forest. (F) A close up of an *A. gonocantha* carton nest entrance. Image credit: California Academy of Sciences, CC-BY 4.0 International license.

**Distribution, life history, and ecology**: *Aphaenogaster* is widespread on Madagascar and constitute important residents of drier regions and open habitats (Fig. 5A). Workers show impressive cooperation in carrying large prey items back to the nest. Their presence in the west of Madagascar can impede the use of bait, such as tuna on white paper cards, along an ant sampling transect. Instead of being attracted to the bait plastered to the paper card, *A. swammerdami* will recruit nest mates to carry the entire card back to the nest. Hence, when you return to check the baits, the cards are gone, only to be found stuffed into the entrances of nearby nests.

In Madagascar, *A. swammerdami* disperses the seeds of *Commiphora guillaumini* (family Burseraceae) in the dry deciduous forests of the west (Böhning-Gaese, Burkhardt & Schmid, 1996). The fruits of *C*. *guillaumini* are bicolored, with a fleshy red aril and a black seed. Ants carry the seeds into their colony, remove the arils, and discard the seeds undamaged on the refuse pile at the edge of the colony. Birds may be the primary seed disperser in this system, but ants serve as important secondary dispersers (Böhning-Gaese, Gaese & Rabemanantsoa, 1999). In the same forest, Voigt et al. (2002) quantified secondary seed dispersal by ants using rice-baiting experiments. They found *A. swammerdami* to be responsible for significantly higher dispersal rates in the Malagasy dry forest compared to sites in South Africa.

Across the distribution of *Aphaenogaster* in Madagascar, there are species of *Camponotus* that mimic the general form and behavior of *Aphaenogaster* (Fig. 5B): *Camponotus imitator* Forel, 1891, *Camponotus jodina* Rasoamanana et al., 2017, *Camponotus karaha* Rasoamanana et al., 2017, and *Camponotus longicollis* Rasoamanana et al., 2017. These *Camponotus* mimics are often seen foraging with workers of *Aphaenogaster*, but potential benefits for *Camponotus* have not been studied.

Throughout western Madagascar, *A. swammerdami* is known locally for its association with snakes (family Colubridae), most notably *Dromicodryas bernieri*, *A. quadrilineatus*, and *Madagascarophis colubrinus* (Preston-Mafham, 1991; Cadle, 2003). Oral tradition also notes this association between snakes and ants. For example, according to people living in the vicinity of the Bezà Mahafaly protected area, *A. swammerdami* hosts a snake that they call *rembitiky* (mother of ants). The story goes that the ants provide a nest for the snake and feed it during the cool dry season. The snake gets bigger and bigger, while the ants reduce the size of the entrance hole until the snake is no longer able to leave. The ants then eat the fattened snake during the rainy season, when it is supposedly difficult for the insects to forage outside. There is no evidence to support the idea that the ants eat the snakes. *Aphaenogaster swammerdami* builds large, deep nests in the soil, which undoubtedly provide good habitat for large snakes seeking shelter (Figs. 5C–5D). Jono, Kojima & Mizuno (2019) conclude that the association with *M. colubrinus* helps defend the ant host larvae from predation by the blind snake *Madatyphlops decorsei*, a specialized predator of ant larvae. Eastern species such as *A. gonacantha* are occasionally found nesting in the ground but more often nest in hollow cavities in dead wood on or near the ground. Species nesting in wood use carton to modify the nest entrance (Figs. 5E–5F).

### Synopsis of Malagasy *Aphaenogaster swammerdami* group species

*bressleri* Csősz & Fisher **sp. n.**

*gonacantha* (Emery, 1899)
*makay* Csősz & Fisher **sp. n.**
*sahafina* Csősz & Fisher **sp. n.**
*swammerdami* Forel, 1886
*= swammerdami clara* Santschi, 1915 **syn. n**
*= swammerdami curta* Forel, 1891 **syn. n**
*= swammerdami spinipes* Santschi, 1911 **syn. n**

**Taxonomic comment**: One species described from Madagascar, *Aphaenogaster friederichsi* Forel, is excluded from the *swammerdami* group and placed in the *A. subterranea* group and synonymized with *A. subterranea* Latreille

*Aphaenogaster friederichsi* Forel, 1918b: 151. Two syntype workers, MADAGASCAR: Diego Suarez (Antsiranana), (K. Friedrichs); CASENT0101054, CASENT0101054; MHNG [examined]. [Combination in Aphaenogaster (Attomyrma): Emery, 1921f: 57.] Junior synonym of *A. subterranea* Latreille **syn. nov.**

*A. friederichsi is* indistinguishable from *A. subterranea* Latreille based on the redescription and neotype designation by Galkowski, Aubert & Blatrix (2019). *A. friederichsi* was described based on two syntype works collected in Diego Surarez, a port city in the North of Madagascar. Repeated collections in the north of Madagascar and in and around Diego Surarez did not uncover this species. The specimens described by Forel could represent a temporarily established introduction of *A. subterranea* or mislabeled specimens of *A. subterranea* collected from Europe. *A. friederichsi* is neither considered native to Madagascar nor established in the region.

### Key to *Aphaenogaster* workers in the Malagasy Region

**Figure 6:**
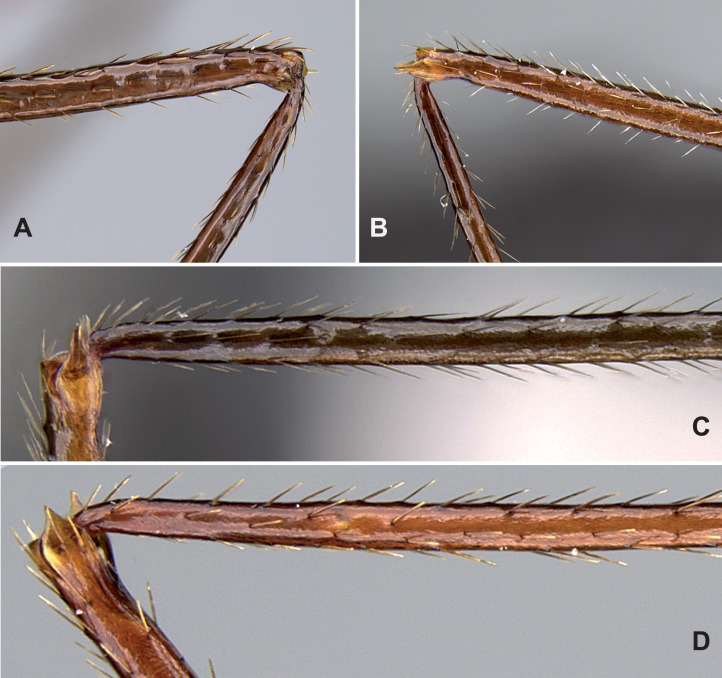
Diagnostic characters for *Aphaenogaster* workers. (A) Lateral view of a rounded apical flange of hind femur (https://www.antweb.org/specimenImages.do?code=CASENT0822320). (B) Lateral view of an acute apical flange of hind femur (https://www.antweb.org/specimenImages.do?code=CASENT0094651). (C) Dense, thin, and acute tarsal setae dense on hind leg (https://www.antweb.org/specimenImages.do?code=CASENT0132233). (D) Sparse, thick, blunt, and bristle-like tarsal setae on hind leg (https://www.antweb.org/specimenImages.do?code=CASENT0495063). Image credit: California Academy of Sciences, CC-BY 4.0 International license.

**Figure 7:**
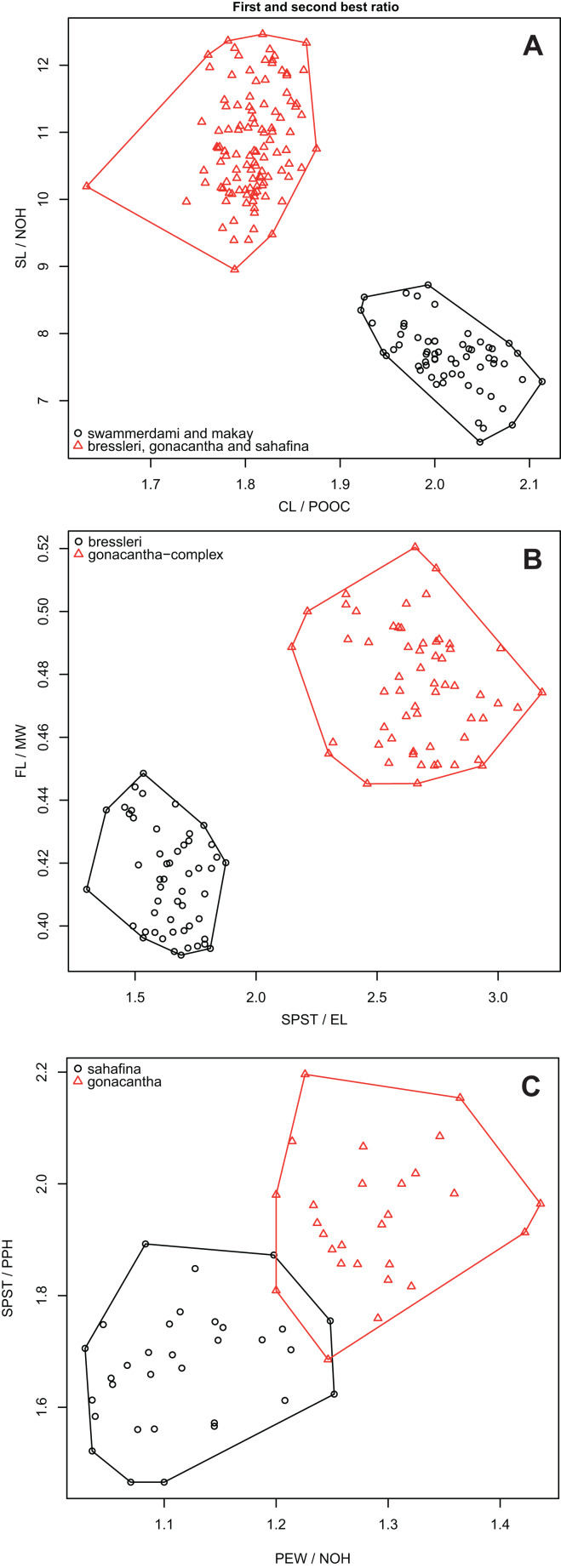
First and second best morphometric ratios. Scatterplots of the two most discriminating ratios (A) between workers of *Aphaenogaster* *swammerdami* complex against *A. gonacantha*, *A. bressleri* **sp. n.**, and *A. sahafina* **sp. n.**; (B) *A. gonacantha* complex (*A. gonacantha* and *A. sahafina* **sp. n.**) and *A. bressleri* **sp. n.**; (C) *A. sahafina* **sp. n. ***and A. gonacantha*. Image credit: California Academy of Sciences, CC-BY 4.0 International license.

1. Apical flange of femur rounded (Fig. 6A). Occipital “neck” shorter, CL/POOC > 1.9 (1.92-2.11) see Fig. 7A. Mesopleuron and mesosoma smooth and shiny. Propodeum feebly rugulose transversally or smooth2
- Apical flanges of femur terminate in an acute, elongate point (Fig. 6B). Occipital “neck” longer, CL/POOC < 1.9 (1.63–1.88) see Fig. 7A. Mesopleuron and mesosoma sculpture variable, smooth to rugose. Propodeum transversally rugose3
2. Propodeal spine very short, tooth-like, SPST/CWb < 0.25 (0.21, 0.23). Neck usually longer, POOC/CWb = 0.88 (0.87, 0.90). The setae on mesosoma dorsum stout, shorter, and with blunt tip*makay*
- Propodeal spine longer, SPST/CWb > 0.25 (0.28, 0.40). Neck often shorter, POOC/CWb = 0.80 (0.70, 0.87). The setae on mesosoma dorsum longer, thinner, and with a sharp tip*swammerdami*
3. Tarsal setae dense, thin, and acute (Fig. 6C). Propodeal spines long, SPST/EL > 2.0 (2.15–3.18) see Fig. 7B4 (*gonacantha*-complex = *gonacantha* and *sahafina*)
- Tarsal setae sparse, thick, and blunt, bristle-like (Fig. 6D). Propodeal spines short, SPST/EL < 2.0 (1.30–1.88) see Fig. 7B*bressleri*
4. In dorsal view, apical flanges of femur divergent, distance between tips of flanges 1.05 to 1.25 times longer than width of distal end of femur. If legs are missing, the PEW/NOH and SPST/PPH ratios help separate this species from *sahafina* (see Fig. 7C)*gonacantha*
- In dorsal view, apical flanges of femur not divergent, distance between tips of flanges equal to width of distal end of femur (upper extreme: tips 1.03 times wider) or convergent. If legs are missing, the PEW/NOH and SPST/PPH ratios help separate this species from *gonacantha* (see Fig. 7C)*sahafina*

### *Aphaenogaster bressleri* Csősz & Fisher sp. n.

**Figure 8:**
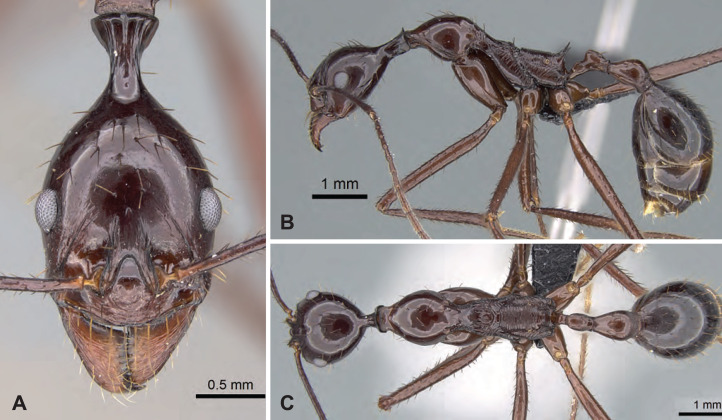
*Aphaenogaster bressleri* holotype worker (CASENT0822137). (A) Head in full-face view, (B) lateral view of the body, (C) dorsal view of the body. Image credit: California Academy of Sciences (https://www.antweb.org/specimenImages.do?code=CASENT0822137), CC-BY 4.0 International license.

Figures 8A–8C, Table 3

*Aphaenogaster bressleri* sp. nov.: urn:lsid:zoobank.org:act:A23F80DA-15E1-4C17-94EA-29DA5CC50D0F;

**Type material. Holotype: MADAGASCAR:** Collection code: **BLF13288b** (CASENT0822137) Toamasina: Reserve Betampona, Camp Vohitsivalana, 37.1 km 338° Toamasina, −17.88667°, 49.2025°, alt 520 m, leg: B.L. Fisher et al., 12_01_2005 (1w, CASC, CASENT0822137);

**Paratypes**: two workers from the same locality (Collection code: **BLF13288**) under CASENT codes: CASENT0067574, CASENT0067682, CASENT0066999, CASENT0822138 (4w, CASC, CASENT0067574);

**Material morphometrically investigated. MADAGASCAR:** Collection code: “**F.C.Andrian.1998xii**” Toamasina: F.C. Andriantantely, −18.695°, 48.81333°, alt 530 m, leg: H.J. Ratsirarson, 12_04_1998 (4w, CASC); Collection code: **ANTC5912** (CASENT0100523) Toamasina: 6.2 km SSE Ambanizana, Be Dinta, −15.66667°, 49.99806°, alt 600 m, leg: V. Razafimahatratra, 11_07_1993 (1w, CASC); Collection code: **BLF00905** (CASENT0175129) Toamasina: 5.3 km SSE Ambanizana, Andranobe, −15.67133°, 49.97395°, alt 425 m, leg: B.L. Fisher, 11_19_1993 (2w, CASC); Collection code: **BLF00906** (CASENT0822143, CASENT0822144) Toamasina: 5.3 km SSE Ambanizana, Andranobe, −15.67133°, 49.97395°, alt 425 m, leg: B.L. Fisher, 11_19_1993 (2w, CASC); Collection code: **BLF00919** Toamasina: 5.3 km SSE Ambanizana, Andranobe, −15.66667°, 49.96667°, alt 600 m, leg: B.L. Fisher, 11_20_1993 (1w, CASC); Collection code: **BLF00926** (CASENT0822135, CASENT0822136) Toamasina: 5.3 km SSE Ambanizana, Andranobe, −15.67133°, 49.97395°, alt 425 m, leg: B.L. Fisher, 11_21_1993 (5w, CASC); Collection code: **BLF08004** (CASENT0048942, CASENT0822140) Toamasina: Montagne d’Anjanaharibe, 18.0 km 21° NNE Ambinanitelo, −15.18833°, 49.615°, alt 470 m, leg: Fisher, Griswold et al., 03_08_2003 (2w, CASC); Collection code: **BLF08138** (CASENT0495063, CASENT0822355, CASENT0495064, CASENT0495065, CASENT0822357) Toamasina: Montagne d’Anjanaharibe, 18.0 km 21° NNE Ambinanitelo, −15.18833°, 49.615°, alt 470 m, leg: Fisher, Griswold et al., 03_08_2003 (6w, CASC); Collection code: **BLF12919** (CASENT0065562, CASENT0822141) Toamasina: Ile Sainte Marie, Forêt Ambohidena, 22.8 km 44° Ambodifotatra, −16.82433°, 49.96417°, alt 20 m, leg: B.L. Fisher et al., 11_22_2005 (2w, CASC); Collection code:

**BLF12934** (CASENT0063309, CASENT0822139) Toamasina: Ile Sainte Marie, Forêt Ambohidena, 22.8 km 44° Ambodifotatra, −16.82433°, 49.96417°, alt 20 m, leg: B.L. Fisher et al., 11_22_2005 (2w, CASC); Collection code: **BLF12985** (CASENT0822142, CASENT0066845) Toamasina: Ile Sainte Marie, Forêt Ambohidena, 22.8 km 44° Ambodifotatra, −16.82433°, 49.96417°, alt 20 m, leg: B.L. Fisher et al., 11_23_2005 (2w, CASC, CASENT0063309); Collection code: **BLF13241** (CASENT0070378) Toamasina: Reserve Betampona, Camp Vohitsivalana, 37.1 km 338° Toamasina, −17.88667°, 49.2025°, alt 520 m, leg: B.L. Fisher et al., 12_01_2005 (1w, CASC);

Collection code: **BLF27668** (CASENT0822356, CASENT0822145, CASENT0822146) Toamasina: Ankerana, −18.40610°, 48.82029°, alt 725 m, leg: B.L. Fisher et al., 2012-01-16, ex rotten log, rainforest (CASENT0822356, 3w, CASC); Collection code: **BLF27719** (CASENT0273507, CASENT0822147, CASENT0822149) Toamasina: Ankerana, −18.4061°, 48.82029°, alt 725 m, leg: B.L. Fisher et al., 01_16_2012 (3w, CASC); Collection code: **BLF28094** (CASENT0274150) Toamasina: Ankerana, −18.4104°, 48.8189°, alt 855 m, leg: B.L. Fisher et al., 01_25_2012 (1w, CASC); Collection code: **BLF28095** (CASENT0274026, CASENT0822148) Toamasina: Ankerana, −18.4104°, 48.8189°, alt 855 m, leg: B.L. Fisher et al., 01_25_2012 (2w, CASC); Collection code:

**HJR121(23)** (CASENT0100521) Toamasina: F.C. Andriantantely, −18.695°, 48.81333°, alt 530 m, leg: H.J. Ratsirarson, 12_04_1998 (1w, CASC); Collection code: Collection code: **HJR121(25)** Toamasina: F.C. Andriantantely, −18.695°, 48.81333°, alt 530 m, leg: H.J. Ratsirarson, 12_04_1998 (1w, CASC); Collection code: Collection code: **HJR122(36)** (CASENT0100522) Toamasina: F.C. Andriantantely, −18.695°, 48.81333°, alt 530 m, leg: H.J. Ratsirarson, 12_07_1998 (1w, CASC); Collection code: **HJR122(37)** Toamasina: F.C. Andriantantely, −18.695°, 48.81333°, alt 530 m, leg: H.J. Ratsirarson, 12_07_1998 (1w, CASC); Collection code: Collection code: **PSW10372** (CASENT0170868) Toamasina: 19 km ESE Maroantsetra, −15.48333°, 49.9°, alt 300 m, leg: P.S. Ward, 04_22_1989 (4w, PSWC).

**Etymology.** The specific epithet is a patronym referring to the late Dr. Barry Lee Bressler, retired physicist, former adjunct professor of physics at Virginia Polytechnic Institute and State University, and amateur naturalist, in recognition of his interest in myrmecology and his support for research on ants. The orthography of a patronym is unchangeable and does not depend on the generic name in which the epithet is used.

**Diagnosis.** Apical flanges of hind femora acute. In lateral view, sides of hind femur taper from midpoint to distal end. Tarsal setae sparse, thick, and blunt, bristle-like. Occipital neck long (POOC/CWb: 0.98 [0.93, 1.06]). Neck constriction smooth and shiny, 2–4 conspicuous carinae visible bilaterally. Median dorsal carina on neck constriction absent, or inconspicuous. Propodeal spine moderately long (SPST/CWb: 0.45 [0.36, 0.56]).

**Description of workers.** Body color dark reddish-brown. Body color pattern: concolorous. Apical flanges of hind femora acute. Tibial setae short, bristle-like. Absolute cephalic size 1,279 µm [1,156, 1,370]. Occipital neck long (POOC/CWb: 0.98 [0.93, 1.06]). Neck constriction smooth and shiny, 2–4 conspicuous carinae visible bilaterally. Median dorsal carina on neck constriction absent, or inconspicuous. Postocular region of cranium smooth and shiny. Frontal lobe distance vs. head width (FL/CWb): 0.32 [0.30, 0.35]. Region between frontal lobes with shallow longitudinal rugae. Scape length vs. head width (SL/CWb): 2.36 [2.25, 2.59]. Antennomere count: 12. Antennal foramen laterally surrounded by one or a few concentric carina(e). Eye length vs. head width (EL/CWb): 0.27 [0.25, 0.30]. Dorsal region of pronotum smooth and shiny. Lateral region of pronotum smooth and shiny. Dorsal region of mesonotum anteriorly smooth, posterior half scabrous. Mesopleuron sculpture scabrous, or vertically costulate. Metapleuron vertically costulate. Dorsal region of propodeum transversally costulate. Propodeal spine moderately long (SPST/CWb: 0.45 [0.36, 0.56]). Propodeal spine tip distance vs. head width (SPTI/CWb): 0.31 [0.17, 0.43]. Petiole width vs. head width (PEW/CWb) 0.26 [0.22, 0.28]. Dorsal region of petiole sculpture smooth and shiny, postero-dorsal surface sometimes dull. Postpetiole width vs. head width (PPW/CWb) 0.40 [0.38, 0.45]. Dorsal region of postpetiole sculpture smooth and shiny.

**Distribution and Biology.** This species is collected in mid-elevation humid forests (20–855 m above sea level) and in humid forests in northeastern Madagascar; its southern range overlaps with *A. sahafina*. According to collection event information, available ground nests of this species can be found under stones or in rotten logs, while workers can be found foraging on the ground and can be sifted from leaf litter.

#### *Aphaenogaster gonacantha* (Emery, 1899)

**Figure 9:**
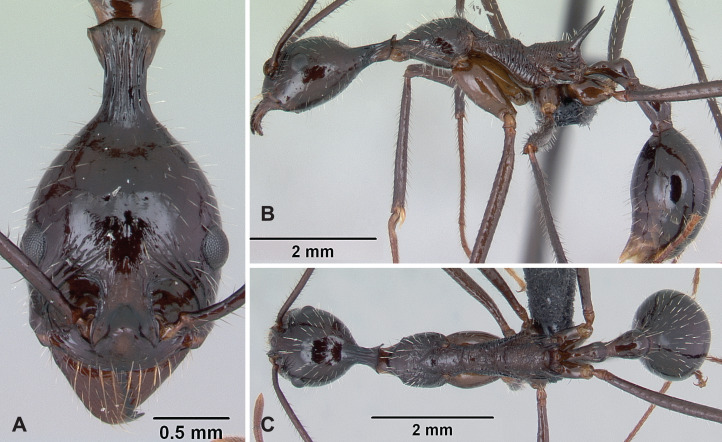
*Aphaenogaster gonacantha* non-type worker (CASENT0107566). (A) Head in full-face view, (B) lateral view of the body, (C) dorsal view of the body. Image credit: California Academy of Sciences (https://www.antweb.org/specimenImages.do?code=CASENT0107566), CC-BY 4.0 International license.

Figures 9A–9C, Table 3

*Ischnomyrmex gonacantha* Emery, 1899: 277

Combination in *Aphaenogaster (Deromyrma)*: Emery, 1915: 71.

**Type material investigated.** *Aphaenogaster gonacantha* Emery, 1899 Syntype series: “Madagascar Antongil Mocquerys”, TYPUS, [−15.4°, 49.8°] (2 syntype workers, CASENT0102038, CASENT0102039 MSNG)

**Material morphometrically investigated. MADAGASCAR:** Collection code: **BLF10998** (CASENT0057287, CASENT0822150) Antsiranana: Forêt Ambanitaza, 26.1 km 347° Antalaha, −14.67933°, 50.18367°, alt 240 m, leg: B.L. Fisher, 11_26_2004 (2w, CASC); Collection code: **BLF11005** (CASENT0107567, CASENT0822152) Antsiranana: Forêt Ambanitaza, 26.1 km 347° Antalaha, −14.67933°, 50.18367°, alt 240 m, leg: B.L. Fisher, 11_26_2004 (2w, CASC); Collection code: **BLF11016** (CASENT0107566, CASENT0822151) Antsiranana: Forêt Ambanitaza, 26.1 km 347° Antalaha, −14.67933°, 50.18367°, alt 240 m, leg: B.L. Fisher, 11_27_2004 (2w, CASC, CASENT0107566); Collection code: **BLF11028** (CASENT0107561, CASENT0822154) Antsiranana: Forêt Ambanitaza, 26.1 km 347° Antalaha, −14.67933°, 50.18367°, alt 240 m, leg: B.L. Fisher, 11_27_2004 (2w, CASC); Collection code: **BLF11096** (CASENT0107368) Antsiranana: Forêt Ambanitaza, 26.1 km 347° Antalaha, −14.67933°, 50.18367°, alt 240 m, leg: B.L. Fisher, 11_28_2004 (1w, CASC); Collection code: **BLF27040** (CASENT0243506, CASENT0822158) Antsiranana: Makirovana forest, −14.104°, 50.03574°, alt 225 m, leg: B.L. Fisher et al., 05_03_2011 (2w, CASC); Collection code: **BLF27044** (CASENT0243596) Antsiranana: Makirovana forest, −14.104°, 50.03574°, alt 225 m, leg: B.L. Fisher et al., 05_04_2011 (1w, CASC); Collection code: **BLF27051** (CASENT0212393, CASENT0212394) Antsiranana: Makirovana forest, −14.104°, 50.03574°, alt 225 m, leg: B.L. Fisher et al., 05_04_2011 (2w, CASC); Collection code: **BLF27191** (CASENT0212356, CASENT0822159) Antsiranana: Makirovana forest, −14.104°, 50.03574°, alt 225 m, leg: B.L. Fisher et al., 05_06_2011 (2w, CASC); Collection code: **BLF32974** (CASENT0375342) Antsiranana: Masoala National Park, −15.32331°, 50.30751°, alt 60 m, leg: B.L.Fisher et al., 03_10_2014 (1w, CASC); Collection code: **BLF32976** (CASENT0376588) Antsiranana: Masoala National Park, −15.32331°, 50.30751°, alt 60 m, leg: B.L. Fisher et al., 03_10_2014 (2w, CASC); Collection code: **BLF33066** (CASENT0375341) Antsiranana: Masoala National Park, −15.32331°, 50.30751°, alt 60 m, leg: B.L. Fisher et al., 03_10_2014 (1w, CASC); Collection code: **BLF08011** (CASENT0049889) Toamasina: Montagne d’Anjanaharibe, 18.0 km 21° NNE Ambinanitelo, −15.18833°, 49.615°, alt 470 m, leg: Fisher, Griswold et al., 03_08_2003 (1w, CASC); Collection code: **BLF08091** (CASENT0497021, CASENT0497022, CASENT0497023, CASENT0497024) Toamasina: Montagne d’Anjanaharibe, 18.0 km 21° NNE Ambinanitelo, −15.18833°, 49.615°, alt 470 m, leg: Fisher, Griswold et al., 03_08_2003 (8w, CASC).

**Etymology**. From angled (*gon*) and spine (*acantha*). We assume that the compound word acts as a noun in apposition and not an adjective since first use was in the masculine genus *Ischnomyrmex* and not subject to gender change if the genus changes.

**Diagnosis.** Apical flanges of hind femora acute. In lateral view, sides of hind femur parallel, not tapering from midpoint to distal end, slight taper occurs just before joint with tibia. Tarsal setae dense, thin, and acute. Occipital neck long (POOC/CWb: 0.97 [0.87, 1.02]). Neck constriction with 7 to 9 conspicuously carinae longitudinally. Median dorsal carina on neck constriction present. Propodeal spine very long (SPST/CWb: 0.70 [0.64, 0.81]).

**Description of workers.** Body color reddish-brown. Body color pattern: head, mesosoma, petiole, and postpetiole reddish-brown, gaster darker. Apical flanges of hind femora acute. Tibial setae long, tapering toward the distal end. Absolute cephalic size 1,453 µm [1,300, 1,580]. Occipital neck long (POOC/CWb: 0.97 [0.87, 1.02]). Neck constriction with 7 to 9 conspicuously carinae longitudinally. Median dorsal carina on neck constriction present. Postocular region of cranium smooth and shiny, or partly dull. Frontal lobe distance vs. head width (FL/CWb): 0.34 [0.31, 0.36]. Region between frontal lobes with shallow longitudinal rugae. Scape length vs. head width (SL/CWb): 2.25 [2.10, 2.38]. Antennomere count: 12. Antennal foramen laterally surrounded by a few concentric carinae. Eye length vs. head width (EL/CWb): 0.25 [0.24, 0.26]. Dorsal region of pronotum scabrous, anterio-median part sometimes smooth. Lateral region of pronotum scabrous. Dorsal region of mesonotum scabrous, or transversally rugose. Mesopleuron sculpture scabrous, or vertically rugose. Metapleuron scabrous, or vertically rugose. Dorsal region of propodeum transversally rugose. Propodeal spine very long (SPST/CWb: 0.70 [0.64, 0.81]). Propodeal spine tip distance vs. head width (SPTI/CWb): 0.45 [0.30, 0.57]. Petiole width vs. head width (PEW/CWb) 0.26 [0.24, 0.28]. Dorsal region of petiole sculpture smooth and shiny, postero-dorsal surface sometimes dull. Postpetiole width vs. head width (PPW/CWb) 0.39 [0.35, 0.42]. Dorsal region of postpetiole sculpture smooth and shiny.

**Distribution and Biology.** This species is collected in humid forests at elevations from 60 m to 470 m above sea level in northern Madagascar. According to collection event information, this species nests in dead branches, rotten logs, and carton nests (Fig. 5F). Foraging workers can also be found on ground or can be sifted from leaf litter. Its distribution overlaps with *A. gonacantha* in its southern range near Antongil Bay.

#### *Aphaenogaster makay* Csősz & Fisher sp. n.

**Figure 10:**
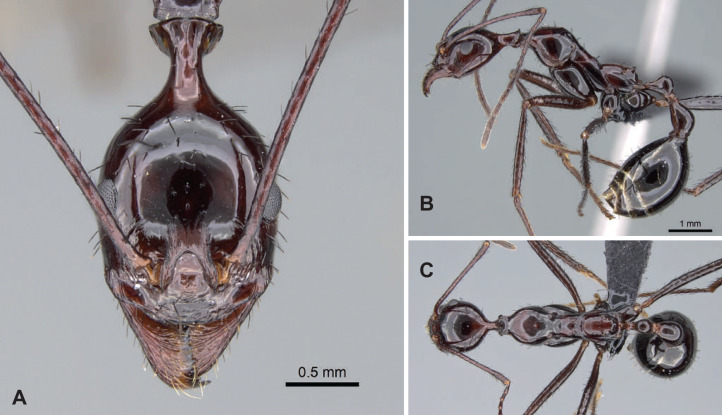
*Aphaenogaster makay* holotype worker (CASENT0208632). (A) Head in full-face view, (B) lateral view of the body, (C) dorsal view of the body. Image credit: California Academy of Sciences (https://www.antweb.org/specimenImages.do?code=CASENT0208632), CC-BY 4.0 International license.

Figures 10A–10C, Table 3

*Aphaenogaster makay* sp. nov.: urn:lsid:zoobank.org:act:08280274-9B3D-4E4F-9E23-477C3ECE1B48;

**Type material. Holotype: MADAGASCAR:** Collection code: **BLF25202** (CASENT0208632) Toliara: Makay Mts., −21.21836°, 45.3106°, alt 510 m, leg: B.L. Fisher et al., 11_24_2010 (1w, CASC, CASENT0208632).

**Paratypes:** Collection code: **BLF25203** (CASENT0210947) Toliara: Makay Mts., −21.21836°, 45.3106°, alt 510 m, leg: B.L. Fisher et al., 11_24_2010 (1w, CASC); Collection code: **BLF25750** (CASENT0205704) Toliara: Makay Mts., −21.25864°, 45.16412°, alt 500 m, leg: B.L. Fisher et al., 12_08_2010 (1w, CASC).

**Etymology.** This species is named after its type locality, the Makay Massif. The orthography of a proper noun is unchangeable and does not depend on the generic name in which the epithet is used.

**Diagnosis.** Apical flange of femur rounded. Tarsal setae dense, thin, and acute. Occipital neck short (POOC/CWb: 0.88 [0.87, 0.90]). Neck constriction smooth, costulae absent or feebly visible. Median dorsal carina on neck constriction absent or feebly visible. Propodeal spine very short (SPST/CWb: 0.22 [0.21, 0.23]).

**Description of workers.** Body color reddish-brown. Body color pattern: head, mesosoma, petiole, and postpetiole reddish-brown, gaster darker. Apical flanges of hind femora blunt, rounded. Tibial setae long, tapering toward the distal end. Absolute cephalic size 1,539 µm [1,418, 1,650]. Occipital neck short (POOC/CWb: 0.88 [0.87, 0.90]). Neck constriction smooth, or feebly visible. Median dorsal carina on neck constriction absent or feeble. Postocular region of cranium smooth and shiny. Frontal lobe distance vs. head width (FL/CWb): 0.33 [0.32, 0.34]. Region between frontal lobes with shallow longitudinal rugae. Scape length vs. head width (SL/CWb): 2.22 [2.15, 2.27]. Antennomere count: 12. Antennal foramen laterally surrounded by one or a few concentric carina(e). Eye length vs. head width (EL/CWb): 0.27 [0.26, 0.28]. Dorsal region of pronotum smooth and shiny. Lateral region of pronotum smooth and shiny. Dorsal region of mesonotum smooth and shiny. Mesopleuron sculpture smooth and shiny. Metapleuron smooth and shiny. Dorsal region of propodeum smooth and shiny, or with shallow transverse rugulae. Propodeal spine very short (SPST/CWb: 0.22 [0.21, 0.23]). Propodeal spine tip distance vs. head width (SPTI/CWb): 0.24 [0.24, 0.25]. Petiole width vs. head width (PEW/CWb) 0.30 [0.29, 0.30]. Dorsal region of petiole sculpture smooth and shiny. Postpetiole width vs. head width (PPW/CWb) 0.42 [0.37, 0.46]. Dorsal region of postpetiole sculpture smooth and shiny.

**Distribution and Biology.** The species was collected from gallery forest with bamboo on sandy soil in the Makay Massif. The bamboo forest was located at the base of cliffs at the headwaters of a canyon stream.

#### *Aphaenogaster sahafina* Csősz & Fisher sp. n.

**Figure 11:**
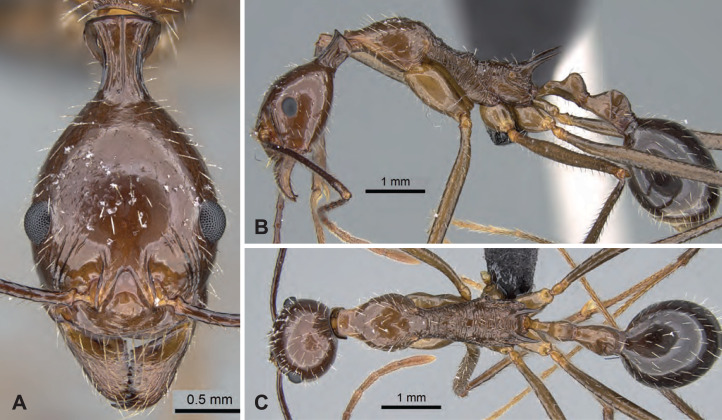
*Aphaenogaster sahafina* holotype worker (CASENT0132233). (A) Head in full-face view, (B) lateral view of the body, (C) dorsal view of the body. Image credit: California Academy of Sciences (https://www.antweb.org/specimenImages.do?code=CASENT0132233), CC-BY 4.0 International license.

Figures 11A–11C, Table 3

*Aphaenogaster sahafina* sp. nov. urn:lsid:zoobank.org:pub:A66CA7F2-7334-40CA-A368-E522BA54ACBF.

**Type material. Holotype: MADAGASCAR:** Collection code: BLF19467 (CASENT0132233) Toamasina: Sahafina forest 11.4 km W Brickaville, −18.81445°, 48.96205°, alt 140 m, leg: B.L. Fisher et al., 12_14_2007 (1w, CASC, CASENT0132233).

**Paratypes**: seven workers and one gyne from the same locality (Collection codes: **BLF19461**, **BLF19467**, **BLF19474**, **BLF19479**) under CASENT codes: CASENT0132230, CASENT0822128, CASENT0822130, CASENT0822133, CASENT0822134, CASENT0822131, CASENT0822132, CASENT0822239 (7w, CASC, CASENT0132239).

**Material morphometrically investigated. MADAGASCAR:** Collection code: **ANTC2685** (CASENT0095660, CASENT0822127) Toamasina: Mahavelona (Foulpointe), −17.66667°, 49.5°, alt, leg: A. Pauly, 04_01_1995 (2w, CASC); Collection code: **ANTC2700** (CASENT0094652, CASENT0094653) Toamasina: Mahavelona (Foulpointe), −17.66667°, 49.5°, alt, leg: A. Pauly, 11_10_1993 (2w, CASC); Collection code: **ANTC2738** (CASENT0095830, CASENT0822123) Toamasina: Mahavelona (Foulpointe), −17.66667°, 49.5°, alt, leg: A. Pauly, 11_01_1995 (2w, CASC); Collection code: **BLF12386** (CASENT0059359, CASENT0822125) Fianarantsoa: 7.6 km 122° Kianjavato, Forêt Classée Vatovavy, −21.4°, 47.94°, alt 175 m, leg: B.L. Fisher et al., 06_06_2005 (2w, CASC); Collection code: **BLF12394** (CASENT0060017, CASENT0060018) Fianarantsoa: 7.6 km 122° Kianjavato, Forêt Classée Vatovavy, −21.4°, 47.94°, alt 175 m, leg: B.L. Fisher et al., 06_06_2005 (2w, CASC, CASENT0060017); Collection code: **BLF12401** (CASENT0060039) Fianarantsoa: 7.6 km 122° Kianjavato, Forêt Classée Vatovavy, −21.4°, 47.94°, alt 175 m, leg: B.L. Fisher et al., 06_06_2005 (1w, CASC); Collection code: BLF12400 (CASENT0060152) Fianarantsoa: 7.6 km 122° Kianjavato, Forêt Classée Vatovavy, −21.4°, 47.94°, alt 175 m, leg: B.L. Fisher et al., 06_06_2005 (1w, CASC); Collection code: **BLF12609** (CASENT0067124, CASENT0822157) Toamasina: Parc National Mananara-Nord, 7.1 km 261° Antanambe, −16.455°, 49.7875°, alt 225 m, leg: B.L. Fisher et al., 11_15_2005 (2w, CASC, CASENT0067124); Collection code: **BLF12668** (CASENT0067123, CASENT0822156) Toamasina: Parc National Mananara-Nord, 7.1 km 261° Antanambe, −16.455°, 49.7875°, alt 225 m, leg: B.L. Fisher et al., 11_16_2005 (2w, CASC); Collection code: **BLF12622** (CASENT0067122, CASENT0822182) Toamasina: Parc National Mananara-Nord, 7.1 km 261° Antanambe, −16.455°, 49.7875°, alt 225 m, leg: B.L.Fisher et al., 11_15_2005 (2w, CASC); Collection code: **BLF12662** (CASENT0067120, CASENT0822160) Toamasina: Parc National Mananara-Nord, 7.1 km 261° Antanambe, −16.455°, 49.7875°, alt 225 m, leg: B.L. Fisher et al., 11_16_2005 (2w, CASC); Collection code: **BLF12668** (CASENT0067123, CASENT0822156) Toamasina: Parc National Mananara-Nord, 7.1 km 261° Antanambe, −16.455°, 49.7875°, alt 225 m, leg: B.L. Fisher et al., 11_16_2005 (2w, CASC); Collection code: **BLF12624** (CASENT0067487, CASENT0822155) Toamasina: Parc National Mananara-Nord, 7.1 km 261° Antanambe, −16.455°, 49.7875°, alt 225 m, leg: B.L. Fisher et al., 11_15_2005 (2w, CASC).

**Etymology.** This species is named after its type locality, Sahafina forest. The orthography of a proper noun is unchangeable and does not depend on the generic name in which the epithet is used.

**Diagnosis.** Apical flanges of hind femora acute. In lateral view, sides of hind femur parallel, not tapering from midpoint to distal end, slight taper occurs just before joint with tibia. Tarsal setae dense, thin, and acute. Occipital neck long (POOC/CWb: 0.99 [0.92, 1.05]). Neck constriction with 5–7 conspicuous carinae longitudinally. Median dorsal carina on neck constriction present. Propodeal spine very long (SPST/CWb: 0.64 [0.55, 0.73]).

**Description of workers.** Body color light brown to brown. Body color pattern: concolorous, or head, mesosoma, petiole and postpetiole brown, gaster darker. Apical flanges of hind femora acute. Tibial setae long, tapering toward the distal end. Absolute cephalic size 1,295 µm [1,064, 1,430]. Occipital neck long (POOC/CWb: 0.99 [0.92, 1.05]). Neck constriction with 5 to 7 conspicuous carinae longitudinally. Median dorsal carina on neck constriction present. Postocular region of cranium smooth and shiny. Frontal lobe distance vs. head width (FL/CWb): 0.34 [0.32, 0.36]. Region between frontal lobes with shallow longitudinal rugae. Scape length vs. head width (SL/CWb): 2.30 [2.17, 2.45]. Antennomere count: 12. Antennal foramen laterally surrounded by one carina or a few concentric carinae. Eye length vs. head width (EL/CWb): 0.25 [0.23, 0.28]. Dorsal region of pronotum medially smooth, lateral and posterior part rugulose or scabrous. Lateral region of pronotum smooth, posteriorly scabrous. Dorsal region of mesonotum scabrous, or transversally rugose. Mesopleuron sculpture scabrous, or vertically rugose. Metapleuron scabrous, or vertically rugose. Dorsal region of propodeum transversally rugose. Propodeal spine very long (SPST/CWb: 0.64 [0.55, 0.73]). Propodeal spine tip distance vs. head width (SPTI/CWb): 0.40 [0.32, 0.58]. Petiole width vs. head width (PEW/CWb) 0.25 [0.21, 0.27]. Dorsal region of petiole sculpture smooth and shiny, postero-dorsal surface sometimes dull. Postpetiole width vs. head width (PPW/CWb) 0.38 [0.34, 0.41]. Dorsal region of postpetiole sculpture smooth and shiny.

**Distribution and Biology.** This species is collected in humid forests between elevations of 140 m and 225 m above sea level in northeastern Madagascar. The distribution overlaps in part with *A. bressleri*. According to collection event information, this species nests in the ground, in rotten logs, and carves out carton nests; in two cases, nests were collected from *Platycerium* stag-horn ferns above the ground. Foraging workers can also be found on the ground or on low vegetation.

#### *Aphaenogaster swammerdami* Forel, 1886

**Figure 12:**
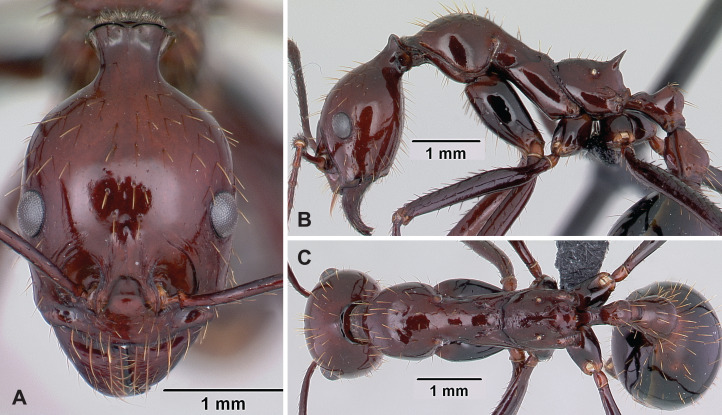
*Aphaenogaster swammerdami* non-type worker (CASENT0178196). (A) Head in full-face view, (B) lateral view of the body, (C) dorsal view of the body. Image credit: California Academy of Sciences; imager: April Nobile (https://www.antweb.org/specimenImages.do?code=CASENT0178196), CC-BY 4.0 International license.

Figures 12A–12C, Table 3

*Aphaenogaster (Ischnomyrmex) swammerdami* Forel, 1886: cvi

Combination in *Aphaenogaster (Deromyrma):* Forel, 1913: 350.

Combination in *Deromyrma*: Santschi, 1915: 250.

*Deromyrma swammerdami* var. *clara* Santschi, 1915c: 250 **syn nov.**

*Aphaenogaster (Ischnomyrmex) swammerdami* var. *curta* Forel, 1891: 169 **syn. nov**.

*Aphaenogaster (Ichnomyrmex) schwammerdami* var. *spinipes* [sic] Santschi, 1911: 123 **syn. nov**.

**Type material investigated.** *Aphaenogaster swammerdami Forel*, 1886 Syntype series: “*A. swammerdami*, Pays Bara (Thosi)” [Madagascar], [−22.3939, 46.1258] (3 syntype workers, CASENT0101073, CASENT0101074, CASENT0101075, MHNG); *Aphaenogaster swammerdami* var. *clara* Santschi, 1915 Syntype series: “*Deromyrma swammerdami* v. *clara*, Baie de Baly.” [Madagascar], [−16.03°, 45.24°] (CASENT0101087, NHMB); *Aphaenogaster swammerdami* var. *curta* Forel, 1891 Syntype series: “*A. swammerdami* var *curta*, Morondaoa, Madagascar”, [−20.3°, 44.3°] (2 syntype workers, CASENT0101066, CASENT0101067, MHNG); *Aphaenogaster swammerdami* var. *spinipes* Santschi, 1911 Syntype worker: “*Deromyrma swammerdami* v. *spinipes*”, [Prov. d’ Ankavandra; Museum Paris, J. Huré 1-98], [−18.77°, 45.29°] (syntype worker, CASENT0101089, NHMB).

**Material morphometrically investigated. MADAGASCAR:** Collection code: **BLF01312** Toliara: southern Isoky-Vohimena Forest, 59 km NE Sakaraha, −22.46667°, 44.85°, alt 730 m, leg: B.L. Fisher, 01_21_1996 (2w, CASC); Collection code: **BLF01474** Toliara: Beza-Mahafaly, 27 km E Betioky, −23.65°, 44.63333°, alt 135 m, leg: B.L. Fisher, 04_23_1997 (6w, CASC); Collection code: **BLF01599** Fianarantsoa: 28 km. SSW Ambositra, Ankazomivady, −20.775°, 47.16833°, alt 1,670 m, leg: B.L. Fisher, 01_09_1998 (2w, CASC); Collection code: **BLF02094** (CASENT0003470) Toliara: Forêt de Petriky, 12.5 km W 272° Tolagnaro, −25.06167°, 46.87°, alt 10 m, leg: B.L. Fisher, 11_22_1998 (2w, CASC); Collection code: **BLF02096** (CASENT0003471) Toliara: Forêt de Petriky, 12.5 km W 272° Tolagnaro, −25.06167°, 46.87°, alt 10 m, leg: B.L. Fisher, 11_22_1998 (1w, CASC); Collection code: **BLF02780** (CASENT0421555, CASENT0421556) Antsiranana: Réserve Spéciale d’Ambre, 3.5 km 235° SW Sakaramy, −12.46889°, 49.24217°, alt 325 m, leg: Fisher, Griswold et al., 01_26_2001 (4w, CASC); Collection code: **BLF03082** (CASENT0421554) Antsiranana: Réserve Spéciale de l’Ankarana, 13.6 km 192° SSW Anivorano Nord, −12.86361°, 49.22583°, alt 210 m, leg: Fisher, Griswold et al., 02_16_2001 (2w, CASC, CASENT0421554); Collection code: **BLF03349** (CASENT0421546, CASENT0421547) Antsiranana: Forêt d’Anabohazo, 21.6 km 247° WSW Maromandia, −14.30889°, 47.91433°, alt 120 m, leg: Fisher, Griswold et al., 03_11_2001 (2w, CASC); Collection code: **BLF03388** (CASENT0421548, CASENT0421551) Antsiranana: Forêt d’Anabohazo, 21.6 km 247° WSW Maromandia, −14.30889°, 47.91433°, alt 120 m, leg: Fisher, Griswold et al., 03_11_2001 (3w, CASC); Collection code: **BLF03519** (CASENT0451462, CASENT0451463) Mahajanga: Parc National d’Ankarafantsika, Ampijoroa Station Forestière, 40 km 306° NW Andranofasika, −16.32083°, 46.81067°, alt 130 m, leg: Fisher, Griswold et al., 03_26_2001 (2w, CASC); Collection code: **BLF03597** (CASENT0484636) Mahajanga: Parc National d’Ankarafantsika, Forêt de Tsimaloto, 18.3 km 46° NE de Tsaramandroso, −16.22806°, 47.14361°, alt 135 m, leg: Fisher, Griswold et al., 04_02_2001 (2w, CASC); Collection code: **BLF03662** (CASENT0484723) Mahajanga: Réserve d’Ankoririka, 10.6 km 13° NE de Tsaramandroso, −16.26722°, 47.04861°, alt 210 m, leg: Fisher, Griswold et al., 04_09_2001 (2w, CASC); Collection code: **BLF04341** (CASENT0078169, CASENT0078170) Mahajanga: Parc National Tsingy de Bemaraha, 2.5 km 62° ENE Bekopaka, Ankidrodroa River, −19.13222°, 44.81467°, alt 100 m, leg: Fisher-Griswold Arthropod Team, 11_11_2001 (2w, CASC); Collection code: **BLF04462** (CASENT0079909, CASENT0079910) Mahajanga: Parc National Tsingy de Bemaraha, 10.6 km ESE 123° Antsalova, −18.70944°, 44.71817°, alt 150 m, leg: Fisher-Griswold Arthropod Team, 11_16_2001 (2w, CASC); Collection code: **BLF04605** (CASENT0473814, CASENT0473845) Toliara: Forêt de Kirindy, 15.5 km 64° ENE Marofandilia, −20.045°, 44.66222°, alt 100 m, leg: Fisher-Griswold Arthropod Team, 11_28_2001 (2w, CASC); Collection code: **BLF04667** (CASENT0001502, CASENT0001503) Toliara: Forêt de Kirindy, 15.5 km 64° ENE Marofandilia, −20.045°, 44.66222°, alt 100 m, leg: Fisher-Griswold Arthropod Team, 11_28_2001 (2w, CASC); Collection code: **BLF04796** (CASENT0429888, CASENT0429889) Toliara: Parc National de Kirindy Mite, 16.3 km 127° SE Belo sur Mer, −20.79528°, 44.147°, alt 80 m, leg: Fisher-Griswold Arthropod Team, 12_06_2001 (2w, CASC); Collection code: **BLF04798** (CASENT0429903, CASENT0429904) Toliara: Parc National de Kirindy Mite, 16.3 km 127° SE Belo sur Mer, −20.79528°, 44.147°, alt 80 m, leg: Fisher-Griswold Arthropod Team, 12_06_2001 (2w, CASC); Collection code: **BLF04864** (CASENT0000973, CASENT0000974) Toliara: Parc National d’Andohahela, Forêt de Manatalinjo, 33.6 km 63° ENE Amboasary, 7.6 km 99° E Hazofotsy, −24.81694°, 46.61°, alt 150 m, leg: Fisher-Griswold Arthropod Team, 01_12_2002 (2w, CASC); Collection code: **BLF04943** (CASENT0001386) Toliara: Parc National d’Andohahela, Forêt d’Ambohibory, 1.7 km 61° ENE Tsimelahy, 36.1 km 308° NW Tolagnaro, −24.93°, 46.6455°, alt 300 m, leg: Fisher-Griswold Arthropod Team, 01_16_2002 (2w, CASC); Collection code: **BLF05239** (CASENT0079228, CASENT0079229) Toliara: Forêt de Mahavelo, Isantoria River, −24.75833°, 46.15717°, alt 110 m, leg: Fisher-Griswold Arthropod Team, 01_28_2002 (2w, CASC); Collection code: **BLF00603** (BLF0603-02) Toliara: Réserve Berenty, −25.01667°, 46.3°, alt 25 m, leg: B.L. Fisher, 12_10_1992 (2w, CASC); Collection code: **BLF06158** (CASENT0002607) Toliara: Forêt de Beroboka, 5.9 km 131° SE Ankidranoka, −22.23306°, 43.36633°, alt 80 m, leg: Fisher-Griswold Arthropod Team, 03_12_2002 (1w, CASC, CASENT0002607).

**Etymology**. The specific epithet is a patronym referring to the Dutch biologist Jan Swammerdam (1637–1680), who carried out the first systematic anatomical studies of social insects. Swammerdam never visited Madagascar. The orthography of a patronym is unchangeable and not depend on the generic name in which the epithet is used.

**Diagnosis.** Apical flange of femur rounded. Tarsal setae dense, thin, and acute. Occipital neck short (POOC/CWb: 0.81 [0.70, 0.90]). Neck constriction smooth, generally 1–3 costulae visible. Median dorsal carina on neck constriction present. Propodeal spine short to moderately long (SPST/CWb: 0.32 [0.21, 0.40]).

**Description of workers.** Body color reddish-brown. Body color pattern: head, mesosoma, petiole and postpetiole reddish-brown, gaster darker. Apical flanges of hind femora blunt, rounded. Tibial setae long, tapering toward the distal end. Absolute cephalic size 1,353 µm [1,030, 1,850]. Occipital neck short (POOC/CWb: 0.80 [0.70, 0.78]). Neck constriction smooth, generally 1–3 costulae visible. Median dorsal carina on neck constriction present. Postocular region of cranium smooth and shiny. Frontal lobe distance vs. head width (FL/CWb): 0.33 [0.29, 0.35]. Region between frontal lobes with shallow longitudinal rugae. Scape length vs. head width (SL/CWb): 1.92 [1.62, 2.16]. Antennomere count: 12. Antennal foramen laterally surrounded by one or a few concentric carina(e). Eye length vs. head width (EL/CWb): 0.27 [0.22, 0.30]. Dorsal region of pronotum smooth and shiny. Lateral region of pronotum smooth and shiny. Dorsal region of mesonotum smooth and shiny. Mesopleuron sculpture smooth and shiny. Metapleuron smooth and shiny. Dorsal region of propodeum smooth and shiny, or with shallow transverse rugulae. Propodeal spine short to moderately long (SPST/CWb: 0.33 [0.28, 0.40]). Propodeal spine tip distance vs. head width (SPTI/CWb): 0.26 [0.16, 0.35]. Petiole width vs. head width (PEW/CWb) 0.26 [0.23, 0.30]. Dorsal region of petiole sculpture smooth and shiny. Postpetiole width vs. head width (PPW/CWb) 0.40 [0.35, 0.46]. Dorsal region of postpetiole sculpture smooth and shiny.

**Distribution and Biology.** This species is widely distributed in dry forests, coastal forests, and spiny forests as well as in cultivated lands of the Western and central highlands of Madagascar. This species typically occurs at lower altitudes between 10 and 200 m above sea level but has been found up to 1,670 m in elevation in the Central Highlands. This species nests in the ground, while foraging workers can also be found on the ground or on low vegetation.

Dittmann, Dammhahn & Kappeler (2014) investigated the density, size, and feeding ecology of colonies at three different sites within the Kirindy Forest (CNFEREF) in central western Madagascar. They observed that the home ranges of *A. swammerdami* extended more or less circularly around the nest entrance and were established by solitary foragers. Colonies maintain exclusive territories and home ranges do not appear to overlap. Diet consisted mainly of very small items below 1 cm, including insects such as termites, ants, and larvae. They measured colony metrics at three sites. The average diameter of mounds at each site ranged between 80 and 125 cm. The estimated number of workers ranged from 200 to 1,000 workers per colony and the home range for foragers averaged 70–250 m^2^, with the largest colony having a home range of 350 m^2^. In a rice baiting experiment, *A. swammerdami* carried seeds to their colony, with a mean dispersal distance of 4.4 ± 1.5 m (Voigt et al., 2002).

## Supplemental Information

10.7717/peerj.10900/supp-1 Supplemental Information 1 Raw morphometric data of Malagasy Aphaenogaster species.

10.7717/peerj.10900/supp-2 Supplemental Information 2 GenBank accession numbers for sequenced samples.

10.7717/peerj.10900/supp-3 Supplemental Information 3 Nucleotide sequences of the 91 sequenced samples.

## Additional Information and Declarations

### Field Study Permissions

The following information was supplied relating to field study approvals (i.e., approving body and any reference numbers): The following information was supplied relating to field study approvals (i.e., approving body and any reference numbers): Ant samples used in this study comply with the regulations for export and exchange of research samples outlined in the Convention of Biology Diversity and the Convention on International Trade in Endangered Species of Wild Fauna and Flora. For field work conducted in Madagascar, permits to research, collect and export ants were obtained from the Ministry of Environment and Forest as part of an ongoing collaboration between the California Academy of Sciences and the Ministry of Environment and Forest, Madagascar National Parks and Parc Botanique et Zoologique de Tsimbazaza. Authorization for export was provided by the Director of Natural Resources. Approval Numbers: No. 0142N/EA03/MG02, No. 340N-EV10/MG04, No. 69 du 07/04/06, No. 065N-EA05/MG11, No. 047N-EA05/MG11, No. 083N-A03/MG05, No. 206 MINENVEF/SG/DGEF/DPB/SCBLF, No. 0324N/EA12/MG03, No. 100 l/fEF/SG/DGEF/DADF/SCBF, No. 0379N/EA11/MG02, No. 200N/EA05/MG02.

### Data Availability

The following information was supplied regarding data availability: Raw data, including GenBank accession numbers, are available in the Supplemental Files. Material is deposited in the ant collection of the California Academy of Sciences, U.S.A. The 176 specimen accession numbers are available in the Supplemental Files. For AntWeb records that include images, please append the CASENT number at the end of this URL: https://www.antweb.org/specimenImages.do?code= "CASENT#"

### New Species Registration

The following information was supplied regarding the registration of a newly described species: Publication LSID: urn:lsid:zoobank.org:pub:A66CA7F2-7334-40CA-A368-E522BA54ACBF. *Aphaenogaster bressleri* Csősz, Loss & Fisher LSID: urn:lsid:zoobank.org:act:A23F80DA-15E1-4C17-94EA-29DA5CC50D0F. *Aphaenogaster makay* Csősz, Loss & Fisher LSID: urn:lsid:zoobank.org:act:08280274-9B3D-4E4F-9E23-477C3ECE1B48. *Aphaenogaster sahafina* Csősz, Loss & Fisher LSID: urn:lsid:zoobank.org:act:0760D603-E566-4E60-B7DF-BDF340177BCB.
